# Supplementary material for: Evaluation of a Conformationally Constrained Indole Carboxamide as a Potential Efflux Pump Inhibitor in Pseudomonas aeruginosa
Source: Antibiotics (Basel). 2022 May 26;11(6):716. doi: 10.3390/antibiotics11060716 (PMC9220351; doi:10.3390/antibiotics11060716)
Supplement: Supplementary file 1 [file antibiotics-11-00716-s001.zip › antibiotics-1714383-supplementary.pdf]

# Supplemental Materials

**Table S1.** *P. aeruginosa* efflux liability of antibiotics tested in this manuscript.

| Antibiotics | MICs (μg/mL) | | | |
|---|---|---|---|---|
| | K767 (WT) | K1455 (↑*mexAB-oprM*) | K2415 (↑*mexXY-oprM*) | K3698 (Δ*oprM*) |
| Cefpirome | 2 | 8 | 8 | 0.5 |
| Imipenem[#] | 2 | 1 | 1 | 2 |
| Levofloxacin | 0.5 | 4 | 4 | 0.031 |
| Cotrimoxazole | 64 | >256 | 64 | 4 |
| Doxycycline | 32 | ≥256 | 32 | 1 |
| Minocycline | 64 | >256 | 32 | 1 |
| Chloramphenicol | 128 | >256 | 128 | 8 |
| Imipenem[#] | 2 | 1 | 1 | 2 |

* Not a substrate of RND efflux pumps in P. aeruginosa.

**Table S2.** Cumulative susceptibility study of *P. aeruginosa* clinical isolates to TXA09155, levofloxacin and the combination.

| Drug | Cumulative Susceptibility % Strains | | | | | | | | | | | | MIC Range (μg/ml) | MIC$_{50}$ (μg/ml) | MIC$_{90}$ (μg/ml) | % R[1] |
|---|---|---|---|---|---|---|---|---|---|---|---|---|---|---|---|---|
| | >32 | 32 | 16 | 8 | 4 | 2 | 1 | 0.5 | 0.25 | 0.12 | 0.06 | ≤0.03 | | | | |
| **TXA09155** | **100** | **36** | **9** | 6 | 3 | 2 | 2 | - | - | - | - | - | 1 - >32 | >32 | >32 | - |
| **LVX** | 100 | 98 | 93 | 86 | 78 | 68 | 58 | 47 | 14 | 2 | - | - | 0.03 - >32 | 1 | 16 | 32 |
| **LVX/ TXA09155[2]** | 100 | 100 | 100 | 98 | 95 | 92 | 88 | 83 | 78 | 65 | 45 | 13 | 0.03 - 32 | 0.12 | 2 | 8 |

[1] Percent resistant per breakpoints (2); LVX ≤1S 2I ≥4R; [2] TXA09155 concentration: 6.25 μg/ml n: 209; LVX: levofloxacin.

**Table S3.** Cumulative susceptibility study of *P. aeruginosa* clinical isolates to TXA09155, levofloxacin and the combination.

| Drug | Cumulative Susceptibility % Strains | | | | | | | | | | | | MIC Range (μg/ml) | MIC$_{50}$ (μg/ml) | MIC$_{90}$ (μg/ml) | % R[1] |
|---|---|---|---|---|---|---|---|---|---|---|---|---|---|---|---|---|
| | >32 | 32 | 16 | 8 | 4 | 2 | 1 | 0.5 | 0.25 | 0.125 | 0.06 | ≤0.03 | | | | |
| **TXA09155** | **100** | **13** | **9** | 7 | 7 | 6 | 2 | - | - | - | - | - | 0.25 - >32 | >32 | >32 | - |
| **LVX** | 100 | 95 | 89 | 84 | 83 | 78 | 69 | 60 | 27 | 4 | 1 | - | 0.06 - >32 | 0.5 | 32 | 22 |
| **LVX/ TXA09155[2]** | 100 | 98 | 94 | 92 | 89 | 88 | 84 | 81 | 78 | 70 | 54 | 18 | ≤0.03 - >32 | 0.06 | 8 | 12 |

[1] Percent resistant per breakpoints (2); LVX ≤1S 2I ≥4R; [2] TXA09155 concentration: 6.25 μg/ml n: 300; LVX: levofloxacin.

**Table S4.** Cumulative susceptibility percentage of *P. aeruginosa* clinical isolates to TXA09155, levofloxacin and the combination.

| Drug | Cumulative Susceptibility % Strains | | | | | | | | | | | | | MIC Range (μg/ml) | MIC$_{50}$ (μg/ml) | MIC$_{90}$ (μg/ml) | % R[1] |
|---|---|---|---|---|---|---|---|---|---|---|---|---|---|---|---|---|---|
| | 128 | 64 | 32 | 16 | 8 | 4 | 2 | 1 | 0.5 | 0.25 | 0.125 | 0.06 | ≤0.03 | | | | |
| **LVX** | **100** | **91** | **62** | 38 | 29 | 21 | 15 | 9 | 9 | 0 | 0 | 0 | 0 | 0.5 - 128 | 32 | 64 | 85 |
| **LVX/** | 100 | 91 | 62 | 44 | 32 | 21 | 15 | 9 | 9 | 0 | 0 | 0 | 0 | 0.5 - 128 | 32 | 64 | 85 |

| | | | | | | | | | | | | | | | | | |
|---|---|---|---|---|---|---|---|---|---|---|---|---|---|---|---|---|---|
| **MC-04,124[2]** | | | | | | | | | | | | | | | | | |
| **LVX/ TXA01182[2]** | 100 | 100 | 100 | 94 | 76 | 53 | 44 | 29 | 24 | 18 | 15 | 6 | 3 | 0.03 - 32 | 4 | 16 | 56 |
| **LVX/ TXA09155[2]** | 100 | 100 | 100 | 100 | 94 | 76 | 65 | 47 | 41 | 24 | 21 | 18 | 9 | ≤0.03 - 16 | 2 | 8 | 35 |

[1] Percent resistant per breakpoints (2); LVX ≤1S 2I ≥4R; [2] EPI concentration: 6.25 µg/ml n: 34; LVX: levofloxacin.

**Table S5.** Levofloxacin potentiation comparison between TXA09155, TXA01182 and MC-04,124 on multidrug-resistant clinical isolates of *P. aeruginosa*.

| **Strain** | **Levofloxacin MIC (µg/mL), (fold difference)** | | | | **Resistance mechanisms** |
|---|---|---|---|---|---|
| | **No EPI** | **+ MC-04,124 (6.25 µg/mL)** | **+ TXA01182 (6.25 µg/mL)** | **+ TXA09155 (50 µg/mL)** | |
| AR-0229 | 64 | 64, (1) | 8, (8) | 2, (32) | *nalC-G71E, mexR-V126Q, gyrA-T83I, OXA-50, PAO* |
| AR-0230 | 64 | 64, (1) | 16, (4) | 8, (8) | *nalC-G71E, T83I, aac(3)-Id, aadA2, cmlA1, dfrB5, OXA-4, OXA-50, PAO, tet(G), VIM-2* |
| AR-0231 | 64 | 64, (1) | 32, (2) | 16, (4) | *nalC-G71E, gyrA-T83I, aac(6')-Iic, KPC-5, OXA-2, OXA-50, PAO* |
| AR-0232 | 8 | 8, (1) | 1, (8) | 0.5, (16) | *nalC-G71E, mexR-V126Q, gyrA-T83I, aadA6, OXA-50, PAO, strA, strB, sul1, tet©* |
| AR-0233 | 4 | 4, (1) | 0.5, (8) | 0.125, (32) | *nalC-G71E, mexR-V126Q, catB7, OXA-50, PAO* |
| AR-0234 | 8 | 8, (1) | 1, (8) | 0.5, (16) | *nalC-G71E, mexR-V126Q, gyrA-T83I, aadA6, OXA-50, PAO, strA, strB, tet©* |
| AR-0236 | 32 | 16, (2) | 8, (4) | 4, (8) | *nalC-G71E, mexR-V126Q, gyrA-T83I, aadB, aph(3')-Iib, OXA-50, PAO* |
| AR-0239 | 64 | 64, (1) | 8, (8) | 4, (16) | *nalC-G71E, mexR-V126Q, gyrA-T83I, aac(6')-Iia, aadB, aph(3')-Ic, cmlA1, dfrB5, GES-1, OXA-10, OXA-50, strA, strB, tet(G), VIM-11* |
| AR-0240 | 128 | 128, (1) | 16, (8) | 8, (16) | *nalC-G71E, mexR-V126Q, gyrA-T83I, aadA6, OXA-50, PAO, sul1, VIM-2* |
| AR-0241 | 64 | 64, (1) | 16, (4) | 8, (8) | *nalC-G71E, mexR-V126Q, gyrA-T83I, aac(6')-Iic, aadA7, catB7, IMP-1, OXA-101, OXA-50, OXA-9, PAO, sul1* |
| AR-0242 | 16 | 8, (2) | 2, (8) | 1, (16) | *nalC-G71E, gyrA-T83I, aac(3)-Id, aadA2, cmlA1, dfrB5, OXA-4, OXA-50, PAO, VIM-2* |
| AR-0244 | 128 | 128, (1) | 8, (16) | 2, (64) | *nalC-G71E, mexR-V126Q, gyrA-T133H, OXA-50* |
| AR-0245 | 128 | 128, (1) | 32, (4) | 16, (8) | *nalC-G71E, mexR-V126Q, gyrA-T83I, aph(3'), OXA, PAO, sul1, VIM-2* |
| AR-0246 | 64 | 64, (1) | 16, (4) | 8, (8) | *nalC-G71E, mexR-V126Q, gyrA-T83I, aadB, NDM-1, OXA-10, OXA-50, PAO, rmtD2, tet(G), VEB-1* |
| AR-0247 | 2 | 2, (1) | 0.031, (64) | ≤0.008, (256) | *nalC-G71E, mexR-V126Q, gyrA-T83I, OXA-50, PAO* |
| AR-0248 | 16 | 16, (1) | 2, (8) | 0.5, (32) | *nalC-G71E, gyrA-T83I, aac(3)-Id, aadA2, cmlA1, dfrB5, OXA-4, OXA-50, PAO, tet(G), VIM-2* |
| AR-0249 | 64 | 64, (1) | 8, (8) | 2, (32) | *nalC-G71E, gyrA-T83I, aac(3)-Id, aadA2, cmlA1, dfrB5, OXA-4, OXA-50, PAO, tet(G), VIM-2* |
| AR-0250 | 64 | 64, (1) | 16, (4) | 8, (8) | *nalC-G71E, mexR-V126Q, gyrA-T83I, aadB, NDM-1, OXA-10, OXA-50, PAO, rmtD2, tet(G), VEB-1* |
| AR-0252 | 32 | 32, (1) | 2, (16) | 0.5, (64) | *nalC-G71E, mexR-V126Q, gyrA-T83I, aadA1, aadA6, OXA-2, OXA-50, PAO, sul1* |
| AR-0253 | 2 | 2, (1) | 0.125, (16) | 0.031, (64) | *nalC-G71E, mexR-V126Q, gyrA-T83I, catB7, OXA-50, PAO* |

| | | | | | |
|---|---|---|---|---|---|
| AR-0256 | 0.5 | 0.5, (1) | 0.062, (8) | 0.031, (16) | *nalC-G71E, catB7, OXA-50, PAO* |
| AR-0258 | 0.5 | 0.5, (1) | 0.125, (4) | 0.062, (8) | *nalC-G71E, mexR-V126Q, OXA-50* |
| AR-0259 | 0.5 | 0.5, (1) | 0.125, (1) | 0.062, (8) | *nalC-G71E, catB7, OXA-50, PAO* |
| AR-0260 | 64 | 64, (1) | 16, (4) | 8, (8) | *nalC-G71E, gyrA-T83I, catB7, OXA-50, PAO* |
| AR-0261 | 8 | 8, (1) | 0.5, (16) | 0.25, (32) | *nalC-G71E, mexR-V126Q, OXA-50, PAO* |
| AR-0262 | 4 | 4, (1) | 0.25, (16) | 0.063, (64) | *nalC-G71E, catB7, OXA-50, PAO* |
| AR-0264 | 32 | 32, (1) | 4, (8) | 1, (32) | *nalC-G71E, D87Y, OXA-50, PAO* |
| AR-0265 | 64 | 64, (1) | 8, (8) | 4, (16) | *nalC-G71E, gyrA-T83I, aadB, catB7, OXA-50, PAO* |
| AR-0266 | 32 | 32, (1) | 8, (4) | 2, (16) | *nalC-G71E, mexR-V126Q, gyrA-T83I, catB7, OXA-50, PAO* |
| AR-0267 | 32 | 32, (1) | 2, (16) | 0.5, (64) | *nalC-G71E, gyrA-T83I, catB7, OXA-50, PAO* |
| AR-0269 | 32 | 32, (1) | 4, (8) | 2, (16) | *nalC-G71E, mexR-V126Q, gyrA-T83I, catB7, OXA-50, PAO* |
| AR-0270 | 32 | 16, (2) | 2, (16) | 0.5, (64) | *nalC-G71E, gyrA-T83I, catB7, OXA-50, PAO* |
| AR-0271 | 32 | 32, (1) | 8, (4) | 4, (8) | *nalC-G71E, mexR-V126Q, gyrA-T83I, catB7, OXA-50, PAO* |
| AR-0272 | 16 | 16, (1) | 4, (4) | 2, (8) | *nalC-G71E, mexR-V126Q, gyrA-T83I, catB7, OXA-50, PAO* |

The *nalC-G71E* mutation is associated with MexAB-OprM overexpression [46-48]. The *mexR-V126Q* mutation is associated with MexAB-OprM overexpression [49-50]. The *gyrA-T83I or gyrA-T133H* mutations lead to fluoroquinolone resistance [26, 51-52].

**Table S6.** Susceptibility of *P. aeruginosa* mutants resistant to TXA09155 or TXA09155/levofloxacin combination to various antimicrobials.

| Strain | MIC (µg/mL) | | | | | | | | |
|---|---|---|---|---|---|---|---|---|---|
| | TXA | LVX | DXC | CAZ | TGC | PMB | AMK | MEM | AZM |
| **ATCC 27853[#]** | 50 | 1 | 32 | 2 | 8 | 2 | 8 | 1 | 64 |
| **EPIR1S** | 200 | 1 | 16 | 2 | 8 | 4 | 8 | 2 | 64 |
| **EPIR9S** | 100 | 1 | 16 | 2 | 8 | 2 | 2 | 1 | 64 |
| **EPIR20L** | 200 | 1 | 16 | 2 | 8 | 4 | 8 | 2 | 64 |
| **EPIR43** | ND | 0.125 | 1 | 0.125 | 2 | 4 | 1 | 0.125 | 16 |
| **EPIR24L** | ND | ND | ND | ND | ND | ND | ND | ND | ND |

[#] Parent strain; TXA, TXA09155; LVX, levofloxacin; DXC, doxycycline; CAZ, ceftazidime; TGC, tigecycline; PMB, polymyxin B; AMK, amikacin; MEM, meropenem; AZM, azithromycin; ND, not determined.
